# A machine learning approach for predicting high risk hospitalized patients with COVID-19 SARS-Cov-2

**DOI:** 10.1186/s12911-022-02076-1

**Published:** 2022-12-28

**Authors:** Alessio Bottrighi, Marzio Pennisi, Annalisa Roveta, Costanza Massarino, Antonella Cassinari, Marta Betti, Tatiana Bolgeo, Marinella Bertolotti, Emanuele Rava, Antonio Maconi

**Affiliations:** 1 DISIT, Computer Science Institute, Università del Piemonte Orientale, Viale T. Michel, 11, 15121 Alessandria, Italy; 2 AI@UPO, Università del Piemonte Orientale, Vercelli, Italy; 3 Research Laboratory Facility, Research and Innovation Department, Azienda Ospedaliera “SS Antonio e Biagio e Cesare Arrigo”, Alessandria, Italy; 4 Research Training Innovation Infrastructure, Research and Innovation Department, Azienda Ospedaliera “SS Antonio e Biagio e Cesare Arrigo”, Alessandria, Italy; 5 DISIT, Università del Piemonte Orientale, Viale T. Michel, 11, 15121 Alessandria, Italy

**Keywords:** COVID-19, Machine learning, Explainability, Patient risk prediction

## Abstract

**Background:**

This study aimed to explore whether explainable Artificial Intelligence methods can be fruitfully used to improve the medical management of patients suffering from complex diseases, and in particular to predict the death risk in hospitalized patients with SARS-Cov-2 based on admission data.

**Methods:**

This work is based on an observational ambispective study that comprised patients older than 18 years with a positive SARS-Cov-2 diagnosis that were admitted to the hospital Azienda Ospedaliera “SS Antonio e Biagio e Cesare Arrigo”, Alessandria, Italy from February, 24 2020 to May, 31 2021, and that completed the disease treatment inside this structure. The patients’medical history, demographic, epidemiologic and clinical data were collected from the electronic medical records system and paper based medical records, entered and managed by the Clinical Study Coordinators using the REDCap electronic data capture tool patient chart. The dataset was used to train and to evaluate predictive ML models.

**Results:**

We overall trained, analysed and evaluated 19 predictive models (both supervised and unsupervised) on data from 824 patients described by 43 features. We focused our attention on models that provide an explanation that is understandable and directly usable by domain experts, and compared the results against other classical machine learning approaches. Among the former, JRIP showed the best performance in 10-fold cross validation, and the best average performance in a further validation test using a different patient dataset from the beginning of the third COVID-19 wave. Moreover, JRIP showed comparable performances with other approaches that do not provide a clear and/or understandable explanation.

**Conclusions:**

The ML supervised models showed to correctly discern between low-risk and high-risk patients, even when the medical disease context is complex and the list of features is limited to information available at admission time. Furthermore, the models demonstrated to reasonably perform on a dataset from the third COVID-19 wave that was not used in the training phase. Overall, these results are remarkable: (i) from a medical point of view, these models evaluate good predictions despite the possible differences entitled with different care protocols and the possible influence of other viral variants (i.e. delta variant); (ii) from the organizational point of view, they could be used to optimize the management of health-care path at the admission time.

## Background

Machine learning (henceforth ML) methods are nowadays applied to an increasing range of research fields that include industrial applications [1], biology and medicine [2, 3], computer vision [4], self-driving systems [5], natural language processing [6], sentiment analysis [7] and so on. However, many ML approaches, particularly those belonging to the field of deep learning, lack explainability. This may represent a major issue from ethical and judicial points-of-view in scientific fields where the model results may positively or negatively influence the health of human beings. Suggestions may be questioned by medical doctors and life scientists if explanations about the reasons and/or features that have been selected and taken into account by the model are missing.

Methodologies coming from the field of explainable Artificial Intelligence (henceforth AI) provide instead interpretable explanations which are understandable to humans and which can be analyzed, tested, verified and/or refuted using either real experiments and data or other knowledge-driven approaches. Among these, of particular interest are those approaches that produce as outcome models based for example on rules or decision trees, as these models can be directly and easily understood by domain experts (such as medical doctors, biologists, epidemiologists, policy makers etc.) without having any specific background.

Explainable AI methods can be fruitfully applied to unravel the real behavior of complex diseases that entitle a wide range of heterogeneous outcomes, especially in emergencies where decisions must be taken promptly. In this scenario their use as second opinion systems may greatly improve both medical and management decisions.

A clear example of such critical situations is represented by the ongoing COVID-19 pandemic, caused by the Severe Acute Respiratory Syndrome CoronaVirus 2 (SARS-CoV-2). SARS-CoV-2 was first identified in Wuhan, China, in December 2019 [8].

The Coronavirus 2019 Disease (COVID-19) represented a global health emergency since its appearance, so the WHO declared a pandemic on 11 March 2020. To contain the outbreak and reduce its spread numerous countries around the world adopted lockdowns and similar societal restrictions [9], leading to global severe social and economic disruption and recession [10]. As of date, over 507 million confirmed cases and over 4.9 million deaths have been reported since the start of the pandemic [11].

COVID-19 patients suffer from varying symptomatology, differing from mild symptoms to severe illness [12]. The symptomatology includes flu-like symptoms, fever, cough or shortness of breath, sneezing, runny nose, sore throat, vomiting, diarrhea, anosmia and dysgeusia. Conjunctivitis and skin rashes are less common [13]. Many patients are asymptomatic or have only mild symptoms, even if they are able to transmit the virus [14].

Cases can progress for the worse evolving into a severe form with risk of complications, especially respiratory [15], and multi-organ failure, leading to death in the most vulnerable individuals. A prompt COVID-19 diagnosis may include medical history, medical examinations, potential extrapulmonary manifestations, and laboratory and radiologic data [16].

Whereas no specific treatment was available at the beginning of the pandemic, nowadays several medications have been approved in different countries [17, 18] and several experimental treatments are being continuously studied in clinical trials [19]. For example, COVID-19 vaccines are widely credited for their role in reducing the severity and death caused by COVID-19 [20].

However, as there is still a high degree of uncertainty on how the health status of patients affected with SARS-CoV2 evolves, in this study we aim to explore whether explainable AI methods can be fruitfully used to improve the medical management of hospitalized patients suffering from complex diseases such as COVID-19, using the limited set of information available ad admission time.

To this end, we used data collected by the “Azienda Ospedaliera SS Antonio e Biagio e Cesare Arrigo” Hospital in Alessandria, Italy, about patients with a positive COVID-19 diagnosis hospitalized from February 24, 2020 to April 4, 2021 to find out if explainable ML methodologies are able to distinguish between patients at low and high risk of death, only using baseline clinical characteristics available at recovery. In particular, we mainly focused on ML approaches which provide a clear and understandable explanation for medical experts (for an in-depth discussion see [21]).

## Methods

This study was approved by the Institutional Ethics Committee (Comitato Etico Interaziendale Alessandria, protocol number ASO.IRFI.20.03). All study procedures complied with the 1946 Declaration of Helsinki [22], the Good Clinical Practices guidelines [23] and relative updating.

### Study design

The “COVID-19 Registry study” has been designed as an ambispective observational study which includes all consecutive patients older than 18 years, admitted to Alessandria Hospital with a confirmed diagnosis of SARS-CoV-2 infection by reverse-transcriptase polymerase chain reaction (RT-PCR) of a nasopharyngeal swab. Retrospective data of hospitalized patients were retrieved between February 24, 2020 and July 14, 2020. Prospective data has been collected since July 15, 2020 up to May, 31 2021. Patients discharged from the Emergency Department were excluded. The study was approved by the Institutional Ethics Committee (Comitato Etico Interaziendale Alessandria, protocol number ASO.IRFI.20.03).

### Data source

Clinical Study Coordinators of the Alessandria Hospital Clinical Trial Center recorded patients data from electronic medical records system (TrackCare) and paper based medical records into a dedicated electronic case report form (eCRF). A pseudonymised code was used to keep safe patient identity according to clinical study and data protection regulations. eCRFs were created by using the freely available Research Electronic Data Capture (REDCap) platform [24, 25], a web-based software platform for designing clinical and translational research databases. The data-entry is done manually and requires a significant effort in terms of time, involving a delay on its availability.

The “COVID-19 Registry” records different patients’ data, including demographics, admission data, past and proximal medical history, onset symptoms, laboratory data, chest X-ray or CT scan results, complications, performed treatments and outcome. A more detailed description is shown in Table 1. For each patient, we calculated Charlson Comorbidity Index [26] and Glasgow Coma Score [27] when possible.

**Table 1:** COVID-19 registry data description

feature Name	Value Type
*Demographics*	
age	Integer
sex	M/F
residence	text
*Admission data*	
date	date
in-hospital ward	Text
diagnosis	text
vital signs	text
*Past and proximal medical history*	
active cancer in the last 5 years	Yes/No
Metastatic disease	Yes/No
acute myocardial infarction	Yes/No
cerebrovascular disease	Yes/No
chronic heart failure	Yes/No
chronic obstructive pulmonary disease	Yes/No
chronic renal failure	Yes/No
connective tissue disease	Yes/No
deep vein thrombosis	Yes/No
dementia	Yes/No
diabetes with or without chronic complications	Yes/No
dyslipidaemia	Yes/No
hepatitis and HIV infection	Yes/No
hypertension	Yes/No
kidney disease	Yes/No
liver disease	Yes/No
obesity	Yes/No
peptic ulcer disease	Yes/No
peripheral vascular disease	Yes/No
pulmonary embolism	Yes/No
other comorbidities	text
home medications	Text
previous vaccinations	Yes/No
smoke habits	unknown/non-smoker/former smoker/smoker
Charlson Comorbidity $\hbox {Index}$	Integer
Glasgow Coma $\hbox {Score}$	Integer
*Onset symptoms*	
fever	Yes/No
chills	Yes/No
hacking cough	Yes/No
phlegm cough	Yes/No
conjunctivitis	Yes/No
rhinorrhea	Yes/No
headache	Yes/No
muscle pain	Yes/No
fatigue	Yes/No
nausea	Yes/No
vomiting	Yes/No
diarrhea	Yes/No
dyspnea	Yes/No
haemoptysis	Yes/No
haematemesis	Yes/No
ageusia	Yes/No
anosmia	Yes/No
abdominal pain	Yes/No
chest pain	Yes/No
pharyngodynia	Yes/No
other symptoms	Text
*Laboratory*	
hematology	numeric
biochemistry	numeric
blood coagulation	numeric
inflammatory markers	Text/Numeric
*Chest X-ray or CT scan results*	
normal	Yes/No
monolateral or bilateral ground-glass opacity	Yes/No
interstitial involvement	Yes/No
irregular shading	Yes/No
*Complications*	
acidosis	Yes/No
acute heart damage	Yes/No
acute kidney injury	Yes/No
acute respiratory distress syndrome	Yes/No
deep vein thrombosis	Yes/No
heart failure	Yes/No
hemorrhages	Yes/No
hypoproteinemia	Yes/No
pneumonia	Yes/No
pulmonary embolism	Yes/No
respiratory decompensation	Yes/No
respiratory failure	Yes/No
rhabdomyolysis	Yes/No
sepsis and septic shock	Yes/No
*Performed Treatments*	
antibiotics	Yes/No
antifungals	Yes/No
antithrombotic prophylaxis	Yes/No
antivirals	Yes/No
chloroquine/hydroxychloroquine	Yes/No
corticosteroids	Yes/No
extra-corporeal membrane oxygenation	Yes/No
immunoglobulins	Yes/No
non-invasive or invasive mechanical ventilation	Yes/No
oxygen therapy (ECMO)	Yes/No
renal replacement therapy	Yes/No
other treatments in accordance to guidelines or experimental drugs	Yes/No
*Outcome*	hospital discharge/transfer/death

### Data description and preparation

The data provided for this study is composed of two datasets. The first dataset is related to the data recorded at the admission time of all hospitalized patients between February 24, 2020 and December 31, 2020, and approximately refers to the first and the second pandemic waves. This dataset initially contained a total of 1405 patients and has been used as baseline for the training of the ML algorithms we tested so far.

The second dataset is composed of the first 100 cases observed during the third wave, in the course of the spreading of the Delta variant (B.1.617.2), collected from February 15, 2021 to April 4, 2021. This dataset has been used to further test and validate ML techniques trained on the first dataset.

All the patients who did not complete the whole disease treatment inside the same structure and were transferred to other structures during their hospitalization period have been excluded from the analysis. We discarded such patients, because any information about the disease development and the patient conditions after the transfer was no more recorded. Furthermore, in most cases, the transferring of a patient to another hospital was mainly determined by administrative and management reasons (e.g., to decrease the pressure on the hospital) rather than health reasons (i.e. based on the disease evaluation). Consequently, the baseline dataset was reduced to 824 patients. The pre-processing for the second dataset led instead to a total of 71 records.

For what regards the features that were used for the analysis, these are mainly related to the fields available at the admission time. Such features include all the onset symptom attributes, comorbidity attributes, age, sex and Charlson comorbidity index.

There were other potentially interesting features in the COVID-19 Registry observational study. These features include, for example, information about previous vaccinations, smoke habits and the Glasgow Coma Score. However, after careful verification, we found that these fields were either poorly populated (for the Glasgow Coma Score) or set to “unknown” (for the smoke habits and the previous vaccinations) for a high percentage of values. Thus, such features were excluded from the analysis. For what regards laboratory data, this kind of data was typically not available at admission time. Also, it presented various missing fields and inconsistencies. For these reasons we also excluded such data from the analysis. In general, the high percentage of missing data for some fields was due to the elevated number of hospital admissions that, particularly during the first wave, did not always allow to record of all the supplementary information.

**Fig. 1:**
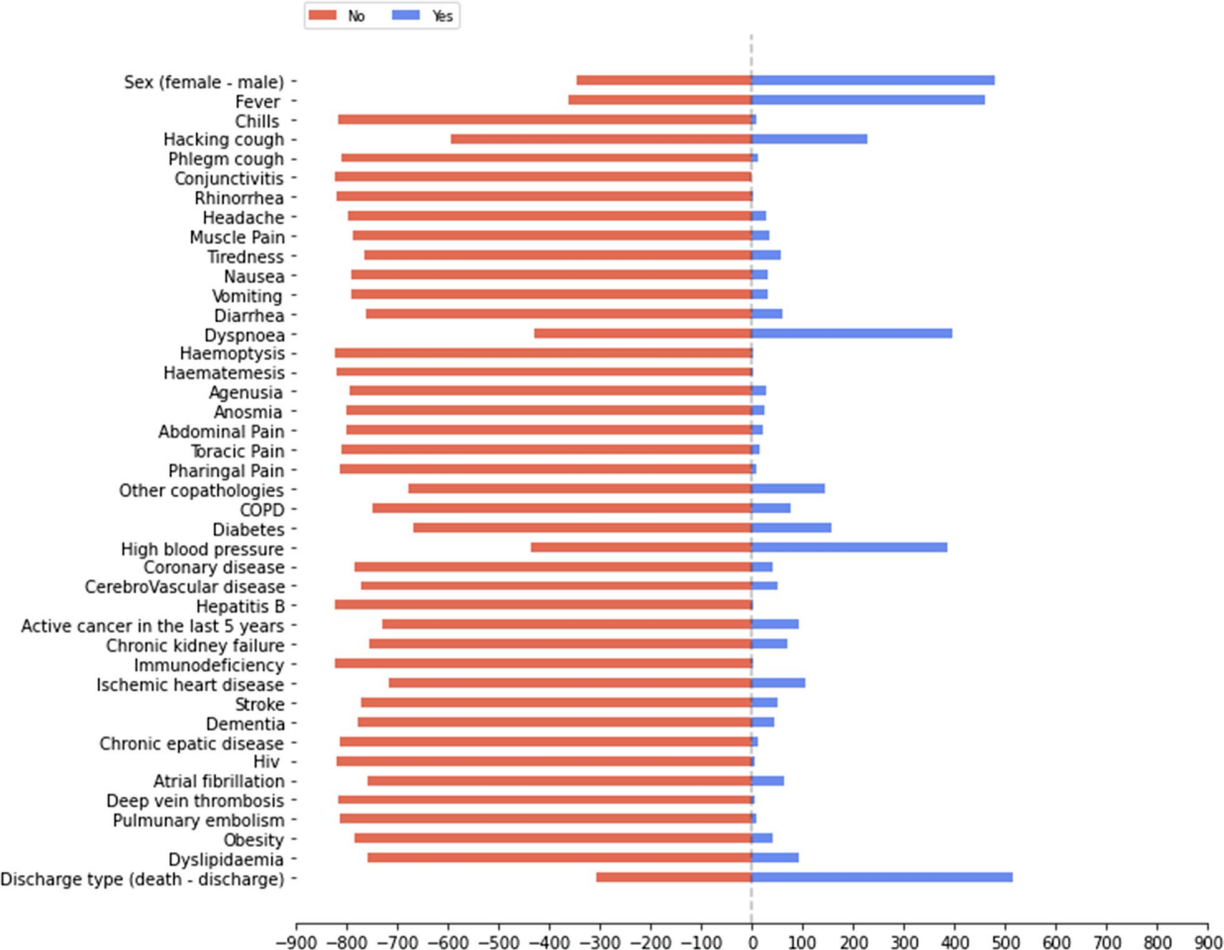
Dichotomic variables distribution. Data refers to the training dataset

**Fig. 2:**
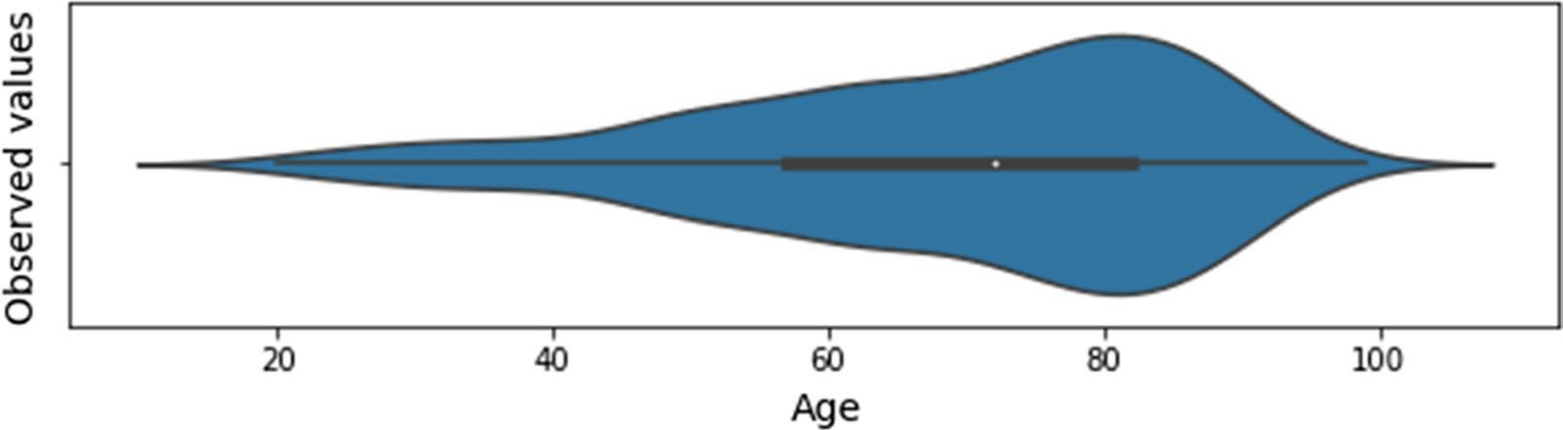
Age distribution. Data refers to the training dataset

**Fig. 3:**
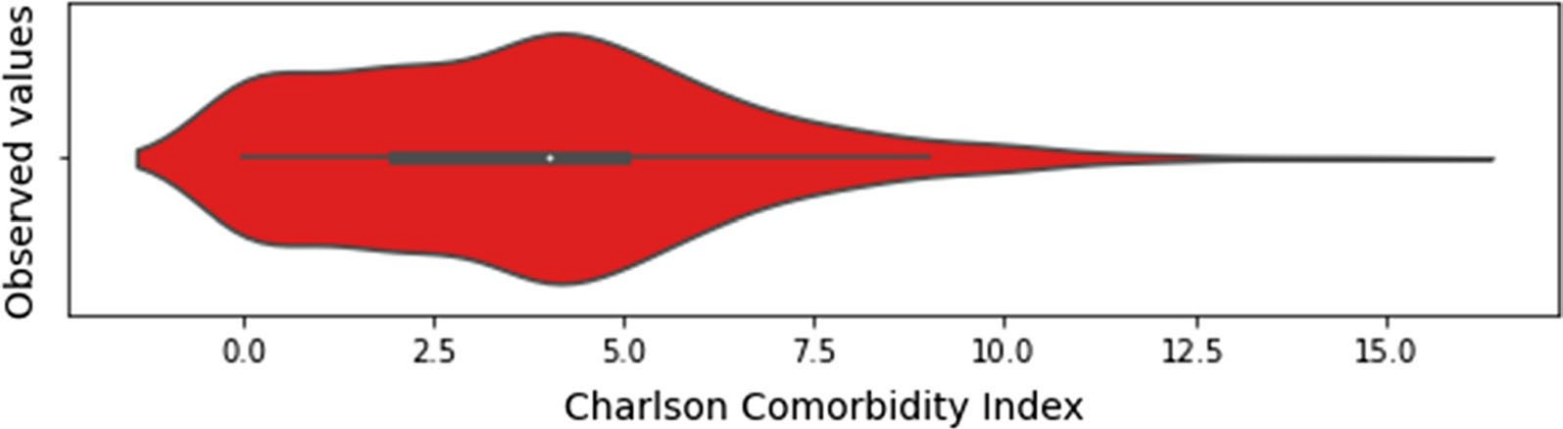
Charlson Comorbidity Index distribution. Data refers to the training dataset

**Fig. 4:**
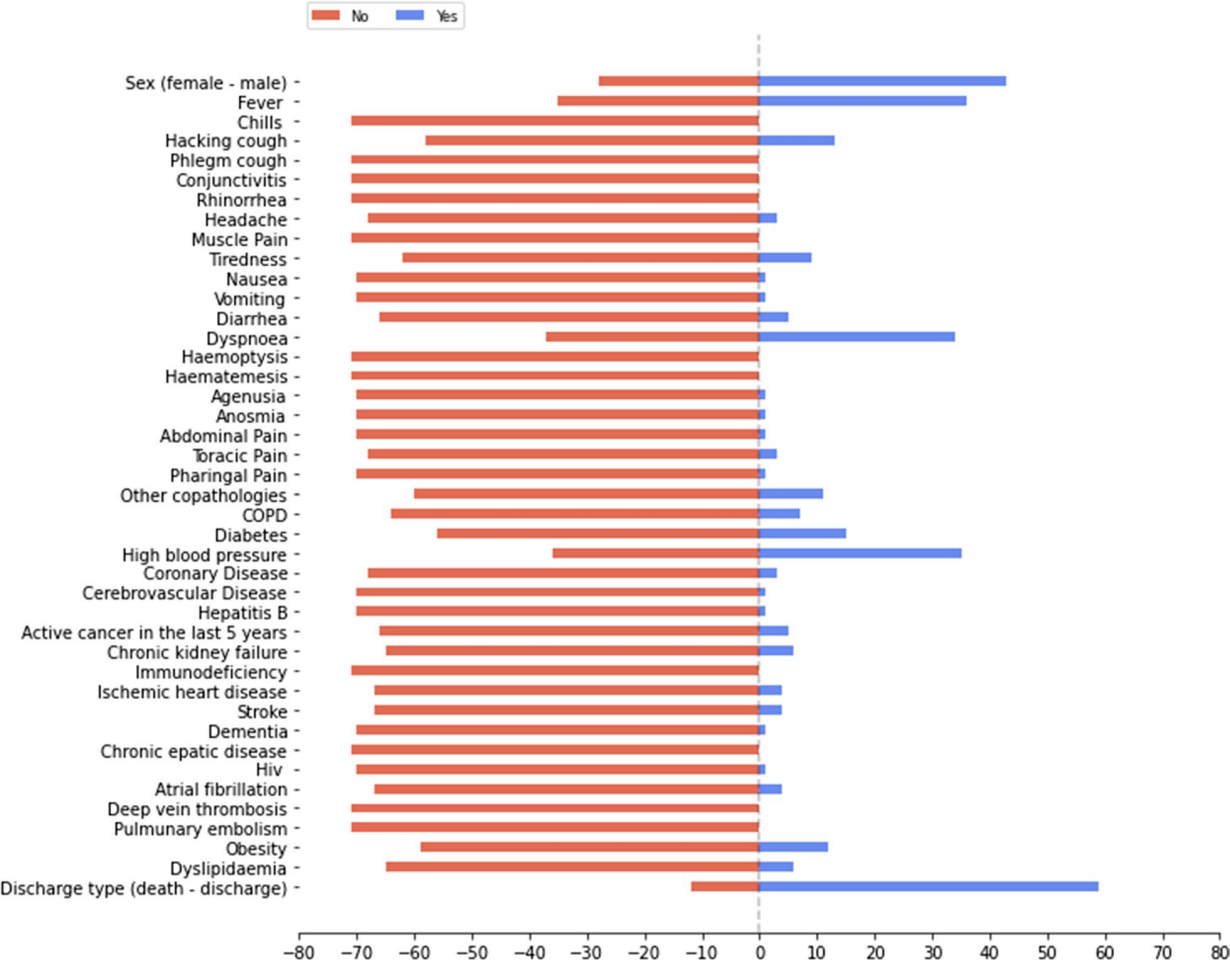
Dichotomic variables distribution. Data refers to the validation dataset

**Fig. 5:**
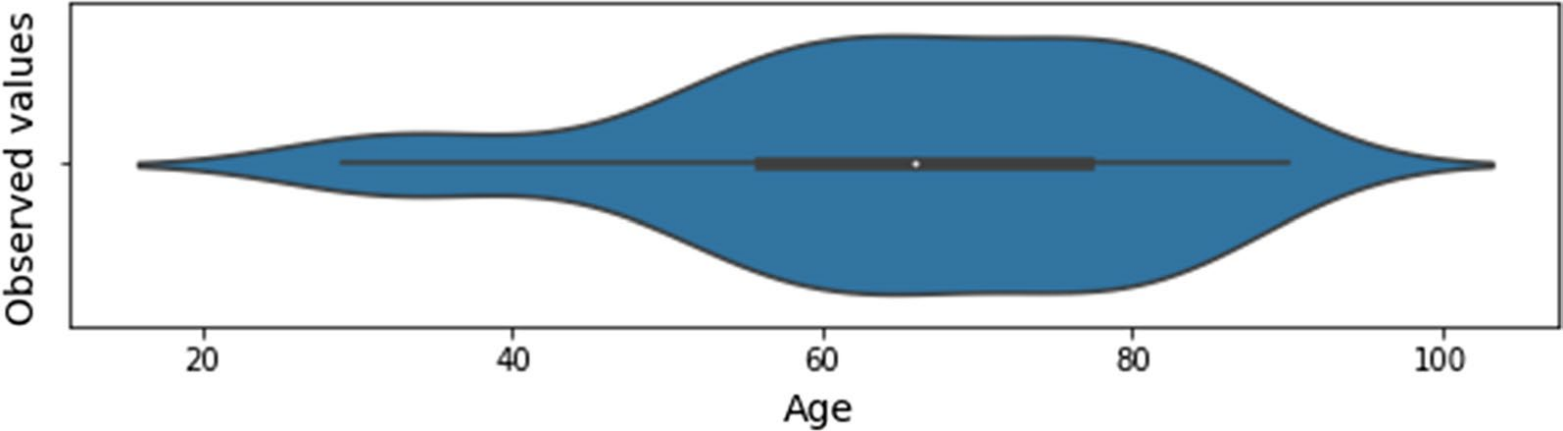
Age distribution. Data refers to the validation dataset

**Fig. 6:**
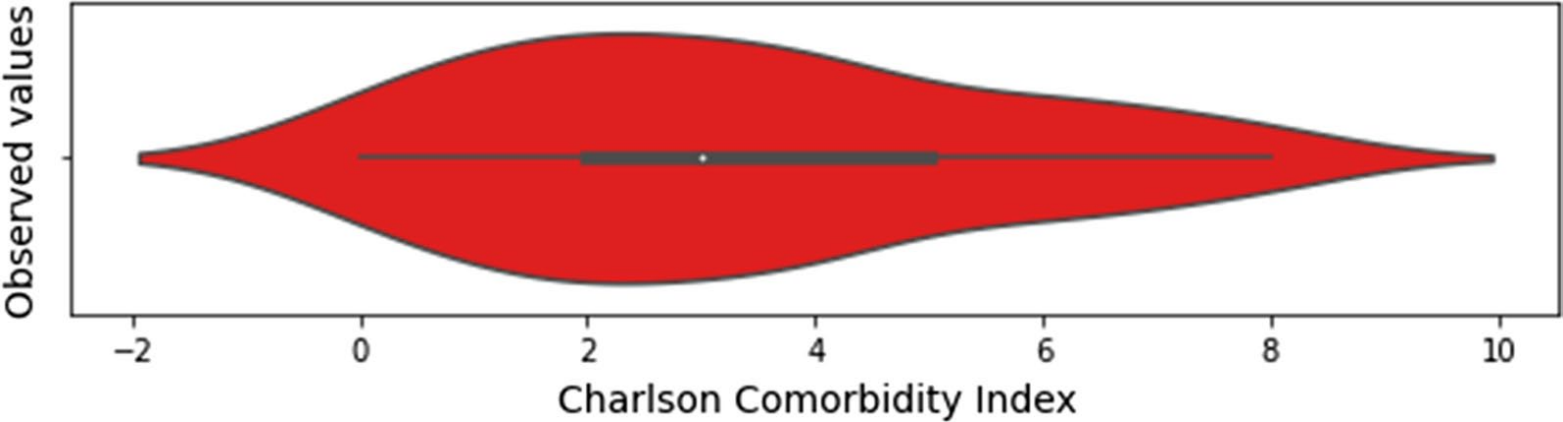
Charlson Comorbidity Index distribution. Data refers to the validation dataset

The list of selected features, whose distribution for the baseline dataset is presented in Figs. 1, 2 and 3, has a total of 43 input features, and one output feature represented by the disease outcome (i.e. discharge type: death or discharge). For what regards this last feature, the number of dead patients is around 37,38%. We underline here that this percentage refers only to the hospitalized patients (i.e., patients with mild to severe symptoms), that completed the whole treatment in the “Azienda Ospedaliera SS Antonio e Biagio e Cesare Arrigo” Hospital, rather than to the total death rate of SARS-CoV-2 patients in the Alessandria province. For what regards the validation dataset, in Figs. 4, 5 and 6 we report the distribution of the 43 input involved features and of the output feature. In such cases, we see that percentage of dead patients drops down to approx. 16,19%, showing how this dataset is somewhat skewed towards discharge outcomes.

### Machine learning methods

The aim of our work was to demonstrate how the use of understandable ML approaches is, at the same time, useful to support medical staff in their work and potentially acceptable thanks to the supplied explanation, which is really important in the medical field. In particular, we focused only on ML models providing an explanation that can be directly understood and then validated by (medical) experts in their application area [21].

The decision of focusing on a specific set of “white box” models only (see *Approaches2* above) was supported by the results of a preliminary study [28], where we tested both white and black box ML approaches on a (reduced) dataset of about 400 patients mainly coming from the first epidemic wave. These results showed that both supervised white and black box approaches performed similarly, with the advantage of the former of providing explainable models. Even if in this preliminary study supervised ML models had very weak performances, for sake of completeness we decided to include them here by using an increased amount of data with respect to that used in [28].

In our study, we have exploited WEKA’s algorithms to perform our experimentation [29]. WEKA is a tool developed at the University of Waikato, New Zealand and it contains tools for data preparation, classification, regression, clustering, association rules mining, and visualization, thus implementing the most common ML algorithms. WEKA is open source software issued under the GNU General Public License.^1^ The ML algorithms have been trained with different configurations. All our experiments on ML models are carried out with 10-fold cross validation on the training set. For sake of simplicity, we report only the configuration with the best results.

First, we tested a set of unsupervised ML models to discover possible regularities in the profiles of the patients. The unsupervised ML models build clusters of patients and provide as output for each cluster a centroid, coupled with a description for it. We tested the following approaches (henceforth *Approaches1*):

- Canopy clustering [30];
- EM clustering [31] (using a free number of clusters and a number of required clusters equal to the number of classes);
- K-means algorithm [32] (K =2);
- Farthest First algorithm [33].
- Hierarchical clustering [34].

Then, we have trained and evaluated different supervised ML models. In particular, we experimented with the following types of classifiers that provide an easily understandable explanation for domain experts (henceforth *Approaches2*):

- Learning decision lists (PART) based on the repeated generation of partial decision trees in a separate-and-conquer manner [35] decision list.
- Decision Tree (DT) classifier, performed with standard C 4.5 algorithm [36];
- Rule classifier (JRIP) performed with standard RIPPER algorithm [37] allowing pruning;
- Random Tree (RT) classifier that considers K randomly chosen attributes at each node performing no pruning [34];
- Reduced Error Pruning Tree (REPTree), a fast decision tree learner that uses the information gain as the splitting criterion, and allows pruning using the error pruning algorithm [34];

Let us point out that the prediction of a new patient is used to identify the class of risk. Thus, a prediction of “In-hospital Death” means that she/he is identified as a high risk patient, otherwise a prediction of “discharged” corresponds to identifying him/her as a low risk patient. Thus, the medical staff can behave accordingly with special attention on high risk patients to better monitor their health status and to readily provide medical interventions when needed. Moreover, having a clear explanation of the classification is fundamental for the medical staff that can directly understand it and evaluate the credibility of the provided suggestions.

For the construction of the ML models the first dataset of patients, described by a total of 43 input features, plus 1 describing the outcome, has been used. Then the second dataset has been used to further test and validate the models.

In the interests of completeness, we finally applied the same experimental framework to some classic ML algorithms (henceforth *Approaches3*) that do not provide an explanation, or that provide an explanation that is not directly understandable and thus usable by medical experts (such as a mathematical function or a Bayesian network). In particular, we considered the ML algorithms analyzed in the preliminary study [28]:

- Bayesian Network (BN) classifier [38] with maximum 1 parent per node,
- Logistic Regression (LR) classifier based on [39] with ridge value 1.0E-8;
- KNN classifier [40] using as number of neighbors to consider in the range from 1 to 9 (we report only the best result, i.e. 8 neighbors);
- SVM classifier with John Platt’s sequential minimal optimization algorithm for training [41].

We also considered the following (black-box) algorithms:

- Voted Perceptron (VP) algorithm by Freund and Schapire [42].
- Random Forest (RF) algorithm [43].
- Adaboost M1 classifier, a statistical classification meta-algorithm [44].
- A bagging classifier to reduce variance. [45]

## Results

824 patients with COVID-19 SARS-Cov-2 were used to train the different ML approaches described above via WEKA.

As previously stated, in the first instance we tried to execute models in *Approaches1* set. Table 2 shows their performances. As it is possible to observe, none of the unsupervised models studied here is able to capture possible regularities in the patients’ profiles, leading in general to very weak performances.

**Table 2:** 10-fold cross validation performances of *Approaches1*

Clustering algorithm	No. of incorrectly clustered instances	% of incorrectly clustered instances
Canopy cluster	267	32.4029%
EM with free no. of clusters	409	49.6359%
EM with 2 clusters	224	27.1845%
Farthest First	278	33.7379%
K-means	315	38.2282%
Hierarchical clustering class	307	37.2573%

Then, we tested the performances of models in *Approaches2* set taking into account the discharge feature as output value (see Table 3). In this scenario, it is instead possible to observe how the models in *Approaches2* set lead in general to far better performances with respect to the ones in *Approaches1* set. Thus, we used the second dataset collected at the beginning of the third pandemic wave as validation dataset for models belonging to *Approaches2* set. Table 4 shows the obtained results for such a scenario. In this case, it is possible to observe a drop in terms of performance for the models in *Approaches2* set.

**Table 3:** 10-fold cross validation performances of *Approaches2*

ML Model	Accuracy	Precision	Recall	F-measure	Roc Area
JRIP	79.1262	0.818	0.791	0.795	0.825
RT	73.7864	0.739	0.738	0.739	0.723
REPTree	78.8835	0.800	0.789	0.791	0.836
PART	75.6068	0.764	0.756	0.759	0.796
DT	79.4903	0.809	0.795	0.798	0.818

**Table 4:** Performances of *Approaches2* on the patient dataset from the begin of the third COVID-19 wave

ML Model	Accuracy	Precision	Recall	F-measure	Roc Area
JRIP	76.0563	0.813	0.761	0.780	0.714
RT	70.4225	0.796	0.704	0.736	0.656
REPTree	69.0141	0.809	0.69	0.726	0.701
PART	74.6479	0.825	0.746	0.772	0.695
DT	67.6056	0.824	0.676	0.716	0.701

Finally, we built and tested the performances of models in *Approaches3* set. Tables 5 and 6 show the performances obtained by 10-fold cross validation and by using the second dataset as test set, respectively.

**Table 5:** 10-fold cross validation performances of *Approaches3*

ML Model	Accuracy	Precision	Recall	F-measure	Roc Area
BN	79.733	0.824	0.797	0.801	0.877
LR	80.9466	0.810	0.809	0.810	0.873
KNN	75.2427	0.752	0.752	0.752	0.813
SVM	79.9757	0.802	0.800	0.801	0.792
VP	69.4175	0.707	0.694	0.649	0.678
RF	79.4903	0.804	0.795	0.797	0.871
Adaboost M1	76.5777	0.785	0.766	0.769	0.837
Bagging	80.3398	0.813	0.803	0.806	0.866

**Table 6:** Performances of *Approaches3* on the patient dataset from the begin of the third COVID-19 wave

ML Model	Accuracy	Precision	Recall	F-measure	Roc Area
BN	70.4225	0.831	0.704	0.740	0.876
LR	76.0563	0.798	0.761	0.776	0.732
KNN	77.4648	0.804	0.775	0.787	0.846
SVM	80.2817	0.843	0.803	0.817	0.749
VP	84.507	0.831	0.845	0.836	0.693
RF	74.6479	0.809	0.746	0.769	0.850
Adaboost M1	69.0141	0.792	0.690	0.724	0.737
Bagging	77.4648	0.833	0.775	0.795	0.775

The performances presented in Table 5 are (in general) quite similar to the ones presented in Table 3. Instead, Table 6 shows that the eight approaches do not produce a homogeneous behaviour in this scenario, with more or less consistent performance variations.

All the ML models built, their configurations for the training and the complete output files of the performance are available at the following link: https://github.com/svezio/CovidStudy.

## Discussion

While unsupervised models (in the *Approaches1* set) seem to fail in catching the disease complexity, supervised ML models are in general able to produce reasonable results. Considering models belonging to the *Approaches2* set, both JRIP and DT seem to overall provide the most solid results, being always first or second for Accuracy, Precision, Recall and F-measure, with only the exception of ROC Area, in which both models are just behind REPtree.

When using the supervised models on the dataset from the third pandemic wave, despite the expected drop, we found that JRIP continues to provide reasonable results, with a precision of 0,813, an F-measure of 0,78, and a Roc Area > 0,7. By taking a deeper look at the confusion matrices, we observed that the majority of incorrect instances refer to patients erroneously classified as potentially dead, while the number of patients incorrectly classified as discharged is in general very low. This suggests that the performance drop is most likely attributable to updated care protocols and/or better management strategies available during the beginning of third wave.

Taking a look at the produced classification model, JRIP is able to bring out a very compact model composed of only 6 rules, as reported in Table 7. Purely by way of example, PART produces a set of 29 rules. By looking at the features selected by JRIP for the definition of the classification rules, age and Charlson comorbidity index represent two of the most important features for profile classification. Also dyspnoea, fever and diabetes may have an important role. These findings are in line with the related literature [46], where older age and comorbidities such as diabetes, hypertension, cardiovascular disease or respiratory diseases have been assessed as major risk factors for moving towards critical or mortal conditions. According to the study, the proportion of diabetes and other comorbidities is statistically significantly higher in critical/mortal conditions compared to non-critical ones. Furthermore, it has been found that clinical manifestations such as shortness of breath, dyspnoea or fever could imply the progression of COVID-19 and are more likely to develop into critical illness or even death [46].

**Table 7:** JRIP produced rules

Rule	Predicted outcome
(dyspnoea = Yes) and (charlsoncomorbidityindex $\leq$ 6)	Death
(charlsoncomorbidityindex $\le$ 4) and (dyspnoea = Yes) (age $\le$ 89)	Death
(charlsoncomorbidityindex $\le$ 4) and (fever = Yes) and (age $\le$ 72)	Death
(age $\le$ 74) and (charlsoncomorbidityindex $\le$ 5)	Death
(diabetes = Yes) and (charlsoncomorbidityindex $\le$ 5)	Death
Else	Discharge

Also, models from the *Approaches3* set are able to provide very solid performances (see Tables 5 and 6 ), and some of them show results that are comparable (if not slightly better) to the best results obtained by models in *Approaches2* for both the tested scenarios (i.e., by using 10-fold cross validation or an external dataset from the third wave).

It is worth noting how BN,^2^ KNN and RF obtain a very remarkable result in terms of ROC Area even when used with the second validation dataset. However, as this second dataset is quite imbalanced (as described in subsection *Data description and preparation*, the use of ROC Area “requires special caution when used with imbalanced datasets” [47]. As suggested in the current literature (see e.g. [47, 48] or for a detailed analysis Chapter 3 in [49]) since ROC Area alone may not be the best informative measure for evaluating the overall model performances, precision and recall scores, and/or other indicators that rely on these (such as F-measure), should be taken instead into consideration for model evaluation as they may be better depict the real model performances. As a consequence of that, we believe that the best approach coming out from the *Approaches3* set is probably represented by SVM.

If we then compare SVM and JRIP (i.e. the best approach among *Approaches2* model set) we will see that the performances of the former seem to be slightly higher than those provided by the latter. However, the small gain of SVM (and in general of *Approaches3* vs. *Approaches2*), if any, remains negligible with respect to the added value, represented by an easily understandable explanation for the domain experts, that the methods in *Approaches2* are able to provide. As already stated, in the medical domain explainability is considered a mandatory feature, which may determine both the acceptability and the applicability of such models.

A similar scenario arises if we compare JRIP with LR (Logistic Regression) from *Approaches3*. Both models, belonging to the field of explainable AI, show very similar performance (see e.g. Tables 4 and 6, respectively). However, the explanation provided by LR is difficult to be directly understandable and usable by medical experts. The LR explanation [50] is an equation that uses all the 43 input attributes. In this equation, there are 43 distinct weights^3^ (i.e. one weight for each attribute), which have a multiplicative effect on the prediction. Thus, the interpretation of attribute relevance is difficult and may not be (in general) so immediate. Furthermore, the real effect of a coefficient on the output cannot be determined independently from the other coefficients even because, for example, the attributes representing rare events (i.e., attributes that are true only for very small portions of the population) may entitle very high coefficients and thus very high odds ratios. However, these attributes result of little relevance in real practical scenarios where such rare events are not so commonly detected. On the other hand, the compactness of the JRIP explanation (i.e., only 6 dichotomous rules) makes the interpretation easier than the LR explanation for the medical experts, as the number of attributes selected for classification is highly reduced (i.e. showing only the relevant attributes for the prediction).

It is worth noting that other studies available in the scientific literature also confirmed the potential use of explainable ML techniques on complex diseases such as Covid-19 [51–53]. These studies also assessed similar findings to those shown in this study, as the prominent role of comorbidities such as diabetes, cardiovascular diseases or the presence of dyspnoea as major risk factors.

## Conclusions

The importance of AI and ML is constantly growing in the last years and their use is rapidly changing the way we approach to and face with real life problems. As a matter of fact, the results in many fields are amazing, but the lack of explainability represents a deal-breaker, especially when the health and safety of human beings are involved. In this scenario (e.g. medical domain), explainable AI techniques should be taken instead into serious consideration.

In our work, we analyzed the performances of ML approaches in the complex medical context of COVID-19. We studied whether ML approaches can predict between low-risk and high-risk COVID-19 hospitalized patients at the admission time. At this step, the early detection of patient risk is crucial, since it can promptly allow appropriate care of high-risk patients. Furthermore, during a pandemic period, such a prediction can improve both organizational and management decisions. Thus, the considered features (i.e. the patient data) are usually limited to ones available at the admission time. In our study, we principally focused on ML approaches which also provide a clear and understandable explanation for domain experts, fostered by the fact that even if a ML model produces good performances, it will hardly be taken into consideration in the medical field without an explanation about its predictions. For the sake of completeness, we also compared such models with other classical ML approaches.

In particular, we have tested 19 ML approaches on COVID-19 patients hospitalized during 2020. While the performances of the all methods in *Approaches1* (i.e. unsupervised ML approaches) were not satisfactory, we showed that methods from *Approaches2* and *Approaches3* entitled quite similar good performances overall.

Let us point out that the methods from the *Approaches2* set can not only be able to correctly discern between low-risk and high-risk in a complex medical disease context and with a limited list of features, but also provide an explanation that is directly usable by medical experts.

The use of patient data from the third COVID-19 wave as test set represents a very important evaluation step, since such patients have not been used to build the models. Models from *Approaches2* set demonstrated able to reasonably perform even in this scenario. From a medical point of view, such a result is also very interesting, because the models produce good predictions despite the possible differences entitled with different care protocols and the possible influence of other viral variants (i.e. delta variant). Moreover, we have compared the results of models from *Approaches2* and *Approaches3* sets. Some methods in *Approaches3* show a small performance advantage, but this gain does not justify their adoption, since explainability is a mandatory feature in the medical domain.

JRIP [37], a propositional rule learner, is one of the approaches showing the best performances overall. Let also us point out that the explanation provided by JRIP is very compact, i.e. a set of six rules with, at most, two or three Boolean conditions. Thus, it is consequently easily understandable and (potentially) usable in real clinical contexts.

Finally, it is worth noting that a possible limitation of this study is given by the fact that the data refers to a period going from the beginning of the pandemic emergency up to the start of the third wave. Virus mutations, as well as improved care protocols and novel treatments (such as antivirals and vaccines), may influence the entire landscape and thus, with a view to a perspective use, models and results should be re-evaluated and refined upon the availability of novel data.

## Data Availability

WEKA produced models and all the obtained results are available at: https://github.com/svezio/CovidStudy.

## Footnotes

1 Available at https://waikato.github.io/weka-wiki/downloading_weka/.

2 Graphical network representation is provided as supplementary material at the following link: https://github.com/svezio/CovidStudy under the folder *Approaches3.*

3 The weights of LR equation are provided in the WEKA output file as supplementary material at the following link: https://github.com/svezio/CovidStudy, under the folder *Approaches3*.
